# Supplementary material for: Genotyping of Le and Af Haplotypes in Dry Pea (Pisum sativam L.) with Field Trials: Short and Semi-Leafless Plants Are Not Always Better in Kazakhstan
Source: Plants (Basel). 2025 Nov 14;14(22):3479. doi: 10.3390/plants14223479 (PMC12656293; doi:10.3390/plants14223479)
Supplement: Supplementary file 1 [file plants-14-03479-s001.zip › plants-3930171-supplementary.pdf]

Arinov, B.; Khassanova, G.; Zailasheva, A.; Kuzbakova, M.; Mussynov, K.; Kipshakbayeva, A.; Jatayev, S.; Tolenova, K.; Sweetman, C.; Jenkins, C.L.D.; Soole K.L., Shavrukov, Y.
Genotyping of *Le* and *Af* haplotypes, and field trials in dry pea (*Pisum sativam* L.): Short and semi-leafless plants not always mean better in Kazakhstan.

Supplementary Table S1. A list of 66 studied pea accessions, their phenotypes and genotypes.

No.	Acces-sion ID	Name	Origin	Plant height	Leaves-tendrils	*Le* ge-notype	*Af* hap-lotype
			**Major pea cultivars grown in Kazakhstan**				
**1**	1	Aksaiskiy usaty55	Russia	medium	semi-leafless	HEX	6
**2**	2	Astronaute	Germany	short	semi-leafless	FAM	5
**3**	3	KASIB	Kazakhstan	tall	semi-leafless	HEX	7
**4**	4	Omskiy neosip.	Russia	tall	regular	HEX	1
**5**	5	Oris	Kazakhstan	medium	regular	HEX	2
**6**	6	Status	Kazakhstan	short	semi-leafless	FAM	8
			**Pea accessions from germplasm collection**				
**1**	3490	New Season	USA	short	regular	FAM	1
**2**	3832	Profi	Denmark	medium	semi-leafless	HEX	8
**3**	4618	IFPI-5208	Argentina	tall	regular	HEX	1
**4**	4859	Split	Serbia	medium	regular	HEX	1
**5**	4969	Ceser	Poland	very tall	regular	HEX	4
**6**	5035	Odesskiy-61	Ukraine	tall	regular	HEX	4
**7**	5051	Nataktaris	Georgia	tall	regular	HEX	1
**8**	5059	Canner Prince	Romania	short	regular	FAM	1
**9**	5094	I/30 Fasciata	Bulgaria	tall	regular	HEX	2
**10**	5111	VII/67-Chlorotica	Bulgaria	very tall	regular	HEX	2
**11**	5120	XV/123-Clarovir.	Bulgaria	short	regular	FAM	3
**12**	5125	XV/890-Supaer.	Bulgaria	short	regular	FAM	4
**13**	5159	Pleven-42	Bulgaria	medium	regular	HEX	4
**14**	5169	Helia	Poland	tall	regular	HEX	2
**15**	5184	Soupa	Australia	medium	regular	HEX	1
**16**	5186	Mukta	Australia	short	semi-leafless	FAM	8
**17**	5245	IG-52457	Morocco	short	semi-leafless	FAM	5
**18**	5404	IFPI-4113	Kazakhstan	medium	regular	HEX	2
**19**	5452	IFPI-4701	Russia	medium	regular	HEX	1
**20**	5502	IFPI-4938	Kazakhstan	tall	regular	HEX	2
**21**	5529	IFPI-5155	Chile	tall	regular	HEX	1
**22**	5543	IFPI-5248	Finland	short	semi-leafless	FAM	8

**23**	5568	WA-1381	Greece	very tall	regular	**HEX**	**1**
**24**	5579	WA-1394	Kazakhstan	tall	regular	**HEX**	**2**
**25**	5672	WA-1489	Turkey	short	regular	**FAM**	**1**
**26**	5781	IFPI-2731	India	very tall	regular	**HEX**	**3**
**27**	5815	IFPI-3627	Nepal	tall	regular	**HEX**	**4**
**28**	5838	Dwarf Champion	Canada	short	regular	**FAM**	**1**
**29**	5840	Elf	UK	short	regular	**FAM**	**1**
**30**	5848	Maple	UK	medium	regular	**HEX**	**4**
**31**	5849	Salome	Denmark	tall	regular	**HEX**	**1**
**32**	5856	X97P19-1	Bulgaria	short	semi-leafless	**FAM**	**6**
**33**	6106	IFPI-4888	Afghanistan	tall	regular	**HEX**	**1**
**34**	6135	IFPI-5061	Cyprus	very tall	regular	**HEX**	**2**
**35**	6160	IFPI-5139	Kazakhstan	tall	regular	**HEX**	**2**
**36**	6167	IFPI-5160	Ethiopia	tall	regular	**HEX**	**1**
**37**	6185	IFPI-5205	Kazakhstan	tall	regular	**HEX**	**3**
**38**	6259	IG-134626	Sweden	short	regular	**FAM**	**4**
**39**	6310	IG-134677	Romania	very tall	regular	**HEX**	**1**
**40**	6326	IG-134693	Poland	short	semi-leafless	**FAM**	**7**
**41**	6570	Kaspa	Australia	short	semi-leafless	**FAM**	**9**
**42**	6588	PS-10128	USA	short	semi-leafless	**FAM**	**8**
**43**	6604	Camera	France	short	regular	**FAM**	**4**
**44**	6605	Booster	France	short	semi-leafless	**FAM**	**6**
**45**	6622	L0358	China	tall	regular	**HEX**	**1**
**46**	6679	IG-52428	Syria	tall	regular	**HEX**	**1**
**47**	6804	IG-52401	Syria	tall	regular	**HEX**	**3**
**48**	6829	IG-124774	New Zealand	short	semi-leafless	**FAM**	**9**
**49**	7101	G0002652	China	very tall	regular	**HEX**	**1**
**50**	7183	G0003746	China	tall	regular	**HEX**	**2**
**51**	7214	G0004001	China	very tall	regular	**HEX**	**2**
**52**	7285	CHNQ04-11-23	China	very tall	regular	**HEX**	**1**
**53**	7301	2002(10)-4	China	short	semi-leafless	**FAM**	**6**
**54**	7327	Arakass	Hungary	very tall	regular	**HEX**	**1**
**55**	7343	JI-2546	Georgia	tall	regular	**HEX**	**3**
**56**	7368	IG-140328	Tajikistan	tall	regular	**HEX**	**1**
**57**	7385	Mangetout Caro.	France	short	semi-leafless	**FAM**	**8**
**58**	7434	PBA Wharton	Australia	short	semi-leafless	**FAM**	**9**
**59**	7442	Diktrom	Australia	short	regular	**FAM**	**1**
**60**	7443	Ebtabi	Australia	very tall	regular	**HEX**	**4**

Supplementary Table S2. Primer design for amplification and further sequencing of Psat5g299720, *Le* gene fragments.

Gene *Le*. AF001219. *Pisum sativum* = LOC127087529

>AF001219.1 Pisum sativum gibberellin 3 beta-hydroxylase (*Le*)

>Psat5g299720.1_Ps_Cameor_v1a. type=mRNA; length = 2725 bp; location = Sequence derived from: chr5LG3. Gene: 567,365,920 – 567,367,724.

AACATTGTCGCCATCTTCTCCATCACCTCTATATATACCAACTTCCCACACAATCACTTTTCTCACT
TCATCTTCATTATTTTCTCTATAGCTACTACTTTTACTCACTATGCCTTCACTCTCCGAAGCCTATA
GAGCACACCCCGTGCACGTTAACCACAAGCACCCTGATTTCAACTCACTTCAAGAACTACCTGAATC
TTACAATTGGACTCACCTTGATGATCACACCCTTATTGATTCCAATAATATTATGAAGGAGAGTACT
ACTACTGTCCCCGTTATTGATCTCAATGACCCTAATGCTTCAAAGCTAATAGGACTTGCATGCAAAA
CATGGGGGGTGTATCAAGTAATGAACCATGGCATCCCCTTAAGCCTTCTTGAGGATATTCAATGGCT
TGGACAAACACTTTTCTCTCTTCCTTCTCACCAAAAACATAAAGCAACTCGTTCCCCCGACGGTGTT
TCGGGATATGGCATCGCTCGTATCTCTTCCTTCTTCCCCAAACTCATGTGGTATGAGGGATTTACTA
TCGTCGGATCACCTCTCGACCATTTTCGAGAACTCTGGCCTCAAGATTATACCAGATTCTGGTAAGT
TACATTGTTAAGAGTCTCTCATCAAATATATATAATTTAATTTTATGATATTAAGTTAAGTCTAAAT
TAAATTTTAAGATATTTCAATCAAATCAATCCTTATGAGTGTGTTAGCGTGTCGTCAATTCAAATTC
AATGCTAATAATAAAAAACCTAAACTAACTAACTATCTCCTCTCTATGAAATCTTATCTTTATTTTT
CACAAATTTATTCTATTTTCCTTTTTAAATAAAATTTAAAATTTGTGGCATTTTTTTGTGGTGACGG
TACTAATGGCCAAACTATATAAGCCCATTTTGTTATCCTATAGTGCACTTGGCAAAGATCACAATGA
AAGCTATGTGTGTGTATATGTATATATCTATTTATGGACCTACACACCCTTTGAAGAATGAAAGGGT
AAACTAATCATGAGAATCCATTGCACCAAATCCACTAGTTTATGTTTGAGATCTCTTAATTATTTAG
TCAACTATGTTTTGATTTTTCCTAACACTCTCCTAATTATATGTGATTTTTTTGGTTTTGTCTTTTC
AGTGATATTGTCGTGCAATATGATGAAACCATGAAAAAGTTAGCAGGAACATTAATGTGTCTAATGT
TGGACTCTCTTGGTATTACAAAGGA**AGATATCAAATGGGCCGGGTCA**AAAGCCCAATTTGAAAAAGC
TTGTGCGGCCCTCCAATTAAACTCCTACCCTAGTTGCCCGGATCCGGATCACGCGATGGGTCTC**R**C
CCCGCACACAGACTCAACATTTTTAACCATCCTATCTCAAAACGACATAAGCGGGTTACAGGTTAAC
CGCGAGGGTTCTGGGTGGATCACGGTTCCACCGCTCCAAGGAGGTCTGGTCGTCAACGTGGGCGACC
TCTTTCATATTTTGTCGAACGGGTTATATCCTAGCGTACTCCATCGAGTTTTAGTGAACCGGACCCG
TCAGAGATTTTCCGTTGCCTATTTATATGGCCCCCCTTCCAATGTAGAGATTTGTCCACATGCAAAA
TTAATAGGCCCAACAAAACCCCCTCTCTATAGGTCAGTGACATGGAATGAGTACCTTGGCACAAAAG
CAAAACATTTCAACAAAGCACTCTCATCTGTTAGACTTTGTACACCTATTAATGGTTTGTTTGATGT
AAACGATTCTAACAAAAATAGTGTCCAAGTGGGCTAAATAGGTATTTCAACTTTTTAATCCTTCCTT
CTTGCATGGTCTATTTTGTACCAACCATATATAAAAAGCCAAAAGGCAATATTGTGAAACGAAACAC
TAGCTATTTTTAAATTTACAATAACTTCAAATTTGGTGGTACAAACAAGAAAGAAATTGAAAGTTCA
AGATTTAGGGTATATACATGGATAAATTTGTTTGGCTCAAAATGGAAGAGGAACCATTATTAATTCA
GAGGATAAGATACATCAAATCAAACAATATTTTTTTAAAAAGTAACTAGTAATGAAAACATAGAGTC
TCACTGTTCTTTCGTTTGTATTTGTAGTGAAAGTGACATAGCAAATTTGTCCAAGTTGAATATATTC
AATTCCACCACAAGACAAACAAAACAACATTTCTTTGGAACTTGATATTTATTTTTTATTT**TATGTG**
GCAACAACCGTACGTGCGCATCTAAAAATTAAAACTGTGTCATTGATCCCCAACTTCACTATGGTAG
TTAATATTAAAGATTAATTGTTCTTGCTAATTTACCGTTTAGGATATTATTTATAGTGAAGTTTTG
TCATTAGAGTTGTCTTGGTGCCCA

[R]=A/G

Primers used for sequencing:

Pisa-Le-Fseq: AGATATCAAATGGGCCGGGTCA 22 bp, 50%GC, Tm=62C
Pisa-Le-Rseq: CACGTACGGTTGTTGCCACATA 22 bp, 50%GC, Tm=62C
(RevCom): TATGTGGCAACAACCGTACGTG
Amplicon size: 995 bp

Supplementary Table S3. ASQ genotyping of *Le* gene: PCR cocktail composition, allele specific primers and universal molecular probes.

ASQ PCR cocktail composition

	Component	For one reaction (µl)	Final concentration
1	DNA template (20 ng/µl)	2.0	4 ng/µl
2	10×SE Buffer (including1.5 mM $MgCl_2$)	1.0	1×
3	dNTP (2 mM)	1.0	0.2 mM
4	AS-primer mix (F1 = 1 µM; F2 = 1 µM; and R = 5 µM)	1.0	0.1+0.1+ +0.5 µM
5	Uni-probe mix (Uni-1 = 1 µM; Uni-2 = 1 µM; and Uni-Q = 10 µM)	0.3	0.03+0.03+ +0.3 µM
6	$MgCl_2$ (25 mM) additional	0.1	1.5+0.25= =1.75 mM
7	SibEnzyme Taq polymerase (5 U/µl)	0.04	0.02 U/µl
8	Water	4.56	
	Total	10.0	

Note: Passive dye ROX was added in Master-mix (1-3 µl) for a few samples or entire microplate when Thermo Fisher qPCR instrument was used. ROX is not required for other qPCR machines, like BioRad.

Amplification program
(1) 95C – 1 min – Initial denaturation
(2) 95C – 10 sec
(3) 59C – 30 sec
(4) Go to step 2 for 10 cycles more – First round of cycles
(5) 95C – 10 sec
(6) 60C – 10 sec
(7) 69C – 45 sec
(8) 54C – 50 sec + Plate read
(9) Go to step 5 for 25 cycles more – Second round of cycles
Note: The duration of the entire program is approximately 1 h and 30 min.

Development of allele-specific primers:

CAAATG**GGCCGGGTCAAAAGCCCA**ATTTGAAAAAGCTTGTGCGGCCCTCCAATTAAACTCCTAC
CCTAGTTGCCCGGATCCGGATCACGCGATGGGTCTC**R****CCCCGCACACAGACTC**AACATTTTTAA
CCATCCTATCTCAAAACGACATAAGCGGGTTACAGGTTAACCGCGAGGGTTCTGGGTGGATCAC

Allele-specific primers:
Allele ‘*a*’ targeting SNP [A] for semi-dwarf genotypes including Astronaute and Cameor:
PsLeSNP1-F1: GAGTCTGTGTGCGGGGTG 18 bp, 67%GC, Tm=61C
(RevCom): CACCCCGCACACAGACTC

Allele '*b*' targeting SNP [G] for tall genotypes including KASIB and Omsky neosip.

PsLeSNP1-F2: AGTCTGTGTGCGGGGCG 17 bp, 71%GC, Tm=60C
(RevCom): CGCCCCGCACACAGACT

PsLeSNP1-R: GGCCGGGTCAAAAGCCCA 18 bp, 67%GC, Tm=61C
Amplicon size: 111 bp

Allele-specific primers with attached 'tails'

PsLeSNP1-F1: GTCCTTGCGAAGGCATCCGAGTCTGTGTGCGGGGTG
PsLeSNP1-F2: GTCCTTGCGAAGGCCAACAGTCTGTGTGCGGGGCG

Common reverse primer:

PsLeSNP1-R: GGCCGGGTCAAAAGCCCA

Universal probes:

Uni1-FAM: FAM-GTCCTTGCGAAGGCATCC
Uni2-HEX: HEX-GTCCTTGCGAAGGCCAAC
Uni-Q: GCCTTCGCAAGGAC**-BHQ1**

Supplementary Table S4. Primer design and regular PCR conditions for analyses of Psat2g173360, *PsPALM1b* gene fragments and surrounding genes near *Afila* locus.

>Psat2g173360-*PsPALM1b*, PsCameor. type=gene; length=1300bp;
location=Sequence derived from: **chr2LG1**: 412,379,762-412,381,061.
TGATAAAATATTTCAACATGTTTACCCTTTTTACATATTTGAATATCTTATGTAAATACTAATAAAT
AGTTTGCCTTCAATGGCGTAGATGCCAAAGGAAAGAATTGTCTTTATTAAGCTATGATCCAACTTCA
AATTTATTAATGGGTGATACATTCGATTTGTTGTTTAAAGTTTAACTCTGTCTTCCTAAATTAATAA
TATCCTCTAGTATATTTAAATAAGCAATTATTTTAATCTTCATTTTCATCCCCCATCACCAATATCT
TTCTATCTTAAATGTCAGTGACAGTACCATGGCTA**CAGATATTGCCCTTCTTTCCATGA**CTACTACT
CAGATCCAAAATTCATCACAATCACAATCAAACCCTAACACCACCAGCACCACCACACCATCACCAT
CAACTTGGATGTGGAACCCTAAACAACAACAACATCAAGAACAAGAAGATGAAGATTCATGGGAGGT
AAGAGCTTTTGCAGAAGACACAAGGAACATGATGAACACAACGTGGCCACCAAGATCCTACACCTGC
ACTTTTTGTAGAAGAGAGTTTCGGTCAGCTCAAGCTCTTGGCGGTCATATGAACGTCCACCGCCGTG
ACCGTGCTCGTCTCCATCAAAATCAACCACCGTTAAATTCCTCCTCTCATCATCCTTCTTCTCCGTT
CATACATATCCCTCCTCAAGAGCTTGTTAATGCTGGATTGTGCCTTTTTTACCATTCACCAAACCCT
AATATTTCTTCCTTCAATGATTCTAATGGAGAATCTCCTTCAACTTTTCTCTCTATCTCATCATCAT
CTTATCCAACAAACAACTTCATGATGCACATGCAACCTTGTTCTCCTCCATCTTTTCATTTTCAGGC
TAATTCAGCTAGAAATTTGATTAACAATAGCATCTCTTCTTTTTCTAACAAACCTACTATCTGCACC
TCCATTGATAATAAGGTTCATGAAATTGAAGAACTCGATCTTGAGCTACGTTTGGGGAACAAGCCAT
CACCGGCATGAAAAATCTATAGCTACATCATAGATATATAGGTGGATGAATTTTTATTTATATTTGA
AGTTATTAATTCATTTTAGCGTGTATGAAAAATATTCAATTATAAATTCCTACAAGTACTCTTATAT
ATTAACTTGTTCTCACTTTTGCAGTGTGTAGTGAAGAAGAAATTAGTGTCCTGTAATTTTCTTAATG
ATTGAGACACCATGTCCA**GCTAGCTTTTTTTATGAAATTGATAGG**ACTATAAATGGA

Primers (5'-3'):
PALM1b-F: CAGATATTGCCCTTCTTTCCATG 23 bp, 43%GC, Tm=61C
PALM1b-R: CCTATCAATTTCATAAAAAAAGCTAGC 27 bp, 30%GC, Tm=61C
(RevCom): GCTAGCTTTTTTTATGAAATTGATAGG
Amplicon size: 948 bp

>Psat2g173880-*PsPALM1a*, PsCameor type=mRNA; length=1559bp.
location=Sequence derived from: **chr2LG1**: 412,901,666-412,903,224.
AATTTATTAAAACCAAATATAATTGTCTTTTACCATTATTAAAATACTACTTTTCTTTTTTAATATA
ACCAGTAACATGTAGTGTCAATCTCACTATGTTCCAACTTCAAATTTATTAATGGGTGATACATTCG
ATTTGATGTTTTAAAGTTTAACTTTGTCTTCCTAAATTAATAATATTCTCTACTATATTTAAATAAG
CAATTATTTTAATCTTCATTTTCATCCCCCATCACCAATATCTTTCTATCTTTCTATCTTAAATGTC
AGCGACAGTACCATGCCTACAGATATTTCCATCACTACTACTCAGATCCAAAATTCATCACAATCAC
AATCTCAATCTCAACCAAACCCTAACATCATCACCACCACCACACCATCATCATCAACTTGGATGTG
GAACCCTAAACAACAACAACATCAAGAACAAGAAGATGAAGATTCATGGGAGGTAAGAGCTTTTGCA
GAAGACACAAGGAACATGATGAACACAACGTGGCCACCAAGATTCTACACCTGCACTTTTTGTAGAA
GAGAGTTCCGGTCAGCTCAAGCTCTTGGCGGTCATATGAATGTCCACCGCCGCGACCGTGCTCGTCT
CCATCAAAATCAACCACCGTTAAACTCCTCCTCTCATCATCCTTCTTCTCCATTCATACATATCCCT
CCTCAAGAGCTTGTTGATGCTGGATTGTGCCTTTTTTACCATTCACCAAACCCTAATATTTCTTCCT
TCAATGATTCTAATGGAGAATCTCCTTCAACTTTTCTCTCTATATCATCATCATCTTATCCAACAAA
CAACTTGATGATGCAAATGCAAATGCAAACTTGTTCTCCTCCATCTTTTCATTTTCAAGCTAATTCA

GCTAGAAATTTGATTAACAATAGCATATCTTCTTTTTCTAACAAACCTGCTATCTGCACCTCCATTA
ATGATAAGGTTCATGAAATTGAAGAACTCGATCTTGAGCTACGTTTGGGGAACAAGCCATCACCGGC
ATGAAAAATCTATAGCTACATCATAGATATATAGATGGATGAATATTTATTTATATTTGAAGTTATT
AATTCATTTAGTGTGTATGAAGAATATTGAATTATAAATTCCTAAAAGTATTCTTATATATTAATT
TGTTCTCTGTCTTTTGCAGCGTGTAGTGAAGAAGAAATTAGTGTCATGTAATTTTCTTAATGATGGA
GACACCATGTCCAGCTATACATGAAATTGATAGCACTATAAATGGATTTTGTTTTTGGTTGGGGTTT
TTGGGGTTAATTTCAATAATTTATTGA

Primers (5'-3'):
PsPALM1a-F: CAGTAACATGTAGTGTCAATCTCAC 25 bp, GC=40%; Tm=63C
PsPALM1a-R: TGCTATCAATTTCATGTATAGCTGG 25 bp, GC=36%; Tm=61C
(RevCom): CCAGCTATACATGAAATTGATAGCA
Amplicon size: 1,172 bp

>Psat2g173920-*PsSA-RNA*, PsCameor.type=mRNA; length=1433bp.
location=Sequence derived from: **chr2LG1**: 412,967,280-412,968,712.
CCCTACGAGAGTAGCAATACTATTTCTCTTATTGCAGTCCAAAGAATCCTTTCACACCAACGGCTAC
TTCTCATTACAAAATGCAACGCGACAATGACATATATCCAAAGTAACCTTTTCTTTCCTTTATAGCC
ACCCAAATAATAATTTTATTCATTTTCTAATTTTCTAATTTTCTAATCCAAATCCATACATAACATA
AATATCATCACACTATTATAAATTTATTTATTCTCAACATTTTAAGTGTTCTAGCAACACAAGGAGC
CACCAACAACATACATATTTCATCCTCTTCAACTACAACCTCGTTTTCCTCAAAAAAAAGCCTACAA
CCTCGTACCTTGCATTTACATATGGATTGTTTGGTGATGCCAGTTTCATTGATCCGGAGAAGATCCA
CAGGCTCACGGTTCGGTTACAAGCCACTAACCGATGATGAGTTGGATCAACAAGATTCCGACAGCCG
GGTGACAGTGGTGGTCGGAAAAGAGAGAAAGGTGTTCCTTGTCGACCCTATCATATTACAAGAGATT
CCGTTTCAGGTTTTGATGGATATCTCTATGAAGAAGAATCCAGAAGAGACAGAGAAGAATCATTTTC
GTTTCACATCAAGTCATCATGATGAGAGAGTTATCTTCGTGGATGTTGATGATATTTTGTTTGAACA
CATGTTGTGGCTTATGTATAATGATGCATCTTCTTTGTTTAAGCTCAATTTGAAAGATATTGTTGAT
TTCTATACTCACGAGGATATGTAATTGTAATAATCTAAATTACAGAGTATTTTCACCCACAACAGAG
GTATTGTATTATGTTAAATTTTCTATTTATGTTCATACTTTAATGT

Primers (5'-3'):
PsSA-RNA-F: GCAGTCCAAAGAATCCTTTCACACC 25 bp, GC=48%; Tm=66C
PsSA-RNA-R: ACACCTTTCTCTCTTTTCCGACCAC 25 bp, GC=48%; Tm=66C
(RevCom): GTGGTCGGAAAAGAGAGAAAGGTGT
Amplicon size: 471 bp

>Psat2g173320-*PsNaOD1*,PsCameor. type=mRNA; length=7456bp;
location=Sequence derived from: **chr2LG1**: 412,297,749-412,305,204.
GTAGTAACCTTACGAACTTTCAAGTATGAAGAAGTAACAGAAAGAAGAAGCGAAAGAGTAACACTAG
TTTCGAAATTCAATGGCTTCAGCAACGACCTTGTTAGAAACACTAGGCAACTTAGAGAGAGATTCAT
TCTTATCTCTTCTCTCCAACCTCATCGGTGAATCCATACACGTGCAAAACAATCCACCGGAACTCAT
TCCTCAAGAGGATAGAGTAGTAAAGCATGTCTTAAACTCACTCCTACCGTTCAGCACCACCACCGGC
GGTGGTCCCTTAATCCTCAACCATGTCACCTATTTTCCTGGCAGAGGTAACCTCATCGTCGAGTATC
CTGGAACCGTTCCCGGCAAGATCCTCTCCTTCGTTGGCTGCCACATGGATGTTGTCACAGCAGATCC
CAAAGATTGGGTAATTATCTATCTATCCATGCATGCATCCATTACTATTATTATGACACATGCATTT

ATATTATTAAGTGTGTTGAATTTGGTGTTGATGTGAAGGATTTTGATCCGTTTACTTTGAGCATCGA
CGGTGATAAGCTTAGGGGTCGTGGAACTACGGATTGTTTGGGACATGTGGCACTTGTTACTGAACTC
ATGAGGAAGCTTGGTGAAACAAAGCCAGATTTGAAATCAACTGTTGTTGCTGTTTTTATAGCGAATG
AGGAGAGTGGTGTTGTAACGGGAGTTGGTATTGATGCACTTGTTCAACATGGTCTTCTCAATAAGCT
AAAAGAAGGTCCCATGTAAGCCATTATTTACTTAAAAAGCTTTTTAATTCGGTCTGATGTCTCGTTA
CAGTGACTTCCACATTGTTGAATAAATTTTAATGTTATAGTTAGTGGATTCCTATTAATAAATTAGT
AGAGAGATTAAGAGTCTGTTGTAGTTGGAAGGTCAAGAGATAGTGACCTATTAATAGTATTTACTAA
TAATTATGTTTTCTAGAATAGCTGAAGTTTTTGAGTTCATATAAAGACCAAGGATGTACTCTAATCA
TAAGATACAGAAGTTAGATTTGCTTCCTTCTGTCAAATTGGCATCAGAGCTAATGGCAAATATGTTG
AATCAAATGTCGTTGTCACGGCTAACGAAGTTAAATTATGAAAACTGAAGTATCCAAATGAAAGCTC
TTCTCGGATCTCTAGACGCGTGGGAGGTGACCAAAGATCGGTTTGAAGAACCAACATATATTGTGGG
ATATACGACAGCTCAAAACAAGGCGTTGAAAGAGACGCGATCGAAGGATAAAACGGCACTATACATG
CTGTTTAGGGCTGTTGATGAATCAGGCTTCGAAAAGATTGTCGGTTCGACTCGATCGAAGGATAAAA
CGGCACTATACATGCTGTTTAGGGCTGTTGATGAATCAGGCTTCGAAAAGATTGTCGGTTCGACTAC
GTCGAAAGAAGCGTGGGACACGCTAGAGAAAGTGTTCAAAGGAGAATATCGAGTAAAGCAAGTTCGA
CTCCAAACTCTTCGTGGCGAATTGGAGAGGATGTAGATGAAGGAGTCAGAAAATGTATCTGACTACA
TCACGCGTGTACAAAAGGTGGTGAACCAACTCACCAGAAATGGCGAAACAGTAATTGATGCACGAGT
TGTTGAAAAGATTTTGAGATCTTTAACAGATAAATTTGAGAATATTGTGTGCGCAATAGAAGAGTCG
AAGGACCTTTCGACGCTCTCAGTCGAAGAGGTCGCTGGTTCCCTCGAAGCACACGAACAACGTAAGA
TGAAAAAGAAGGAAGAAGGAGTAGAGGACGCAAATCAAACCAAGGAGCAAATCAAAGACGAAAAGGT
ACTCTTTTCTCAAAAATTTCGAGGAAGAGGACGTGGTCGTGGAGGACGTGACAGTGGTAGAAGTGGT
AGAGGCAGCAACTTCGAGAGAGGACAGTTGAGCCAGCAAAATTGGCGTGGCAGAGGACGTGGTCAAA

Primers (5′-3′):
PsNaOD1-F: ACGTGCAAAACAATCCACCG 20 bp, GC=50%; Tm=58C
PsNaOD1-R: CGAAGTTGCTGCCTCTACCA 20 bp, GC=55%; Tm=60C
(RevCom): TGGTAGAGGCAGCAACTTCG
Amplicon size: 1,719 bp

Regular PCR mix composition:

Regular PCR was performed in 15 µl volume reactions containing 2 µl of template DNA adjusted to 50 ng/ml, and with the following components in their final concentrations as listed: 1×PCR buffer, 2 mM $MgCl_2$, 0.2 mM each of dNTPs, 0.25 mM of each primer and 1.0 units of GoTaq Flexi DNA polymerase (Promega, USA) in each reaction. PCR was conducted on a Thermal iCycler (Bio-Rad, USA), using a program with the following steps: initial denaturation, 94°C, 2 min; 35 cycles of 94°C for 15 s, 55°C for 15 s, 72°C for 1 min, and a final extension of 72°C for 3 min. PCR products were separated in a 1% agarose gel with adding of 1 kb DNA Ladder (Axygen, Union City, CA USA). Presence/absence of bands were visualised with GelRed (Biotium, Fremont, CA, USA) using a GelDoc imaging system (BioRad, Hercules, CA, USA).
